# Supplementary material for: Complexome profiling of the Chlamydomonas psb28 mutant reveals TEF5 as an early PSII assembly factor
Source: Plant Cell. 2025 Mar 17;37(6):koaf055. doi: 10.1093/plcell/koaf055 (PMC12164590; doi:10.1093/plcell/koaf055)
Supplement: koaf055_Supplementary_Data [file koaf055_supplementary_data.zip › psb28_interactive.zip!/plots/intensity/qualityPlots/peptides/APT1_Cre01.g002050.t1.1.html]

### Cre01.g002050.t1.1

Trivial name: APT1  
SeqCoverage [median]: 12.40  
Mass: 77.32 kDa  
  
 

| Sequence | MissedCleavage | Mass [Da] | wtIntensity | muIntensity | Score |
| --- | --- | --- | --- | --- | --- |
| AAAAAAAAGDSGVGAGAEVEAKEQAK | 1 | 2270.11 | 0.00 | 561.13 | 86.55 |
| AKPGAAQAEAGVAAAAQEEVAK | 1 | 2037.05 | 1006.77 | 0.00 | 66.05 |
| LFSTSGVENPVSR | 0 | 1391.70 | 641.25 | 1614.16 | 138.25 |
| LGQAQAR | 0 | 742.41 | 579.89 | 538.48 | 88.35 |
| MGHDTPLPQPK | 0 | 1219.60 | 855.87 | 0.00 | 64.82 |
|  |  |  | 3083.8 | 2713.8 |  |
